# Supplementary material for: Preharvest Salicylate Treatments Enhance Antioxidant Compounds, Color and Crop Yield in Low Pigmented-Table Grape Cultivars and Preserve Quality Traits during Storage
Source: Antioxidants (Basel). 2020 Sep 6;9(9):832. doi: 10.3390/antiox9090832 (PMC7555001; doi:10.3390/antiox9090832)
Supplement: Supplementary file 1 [file antioxidants-09-00832-s001.pdf]

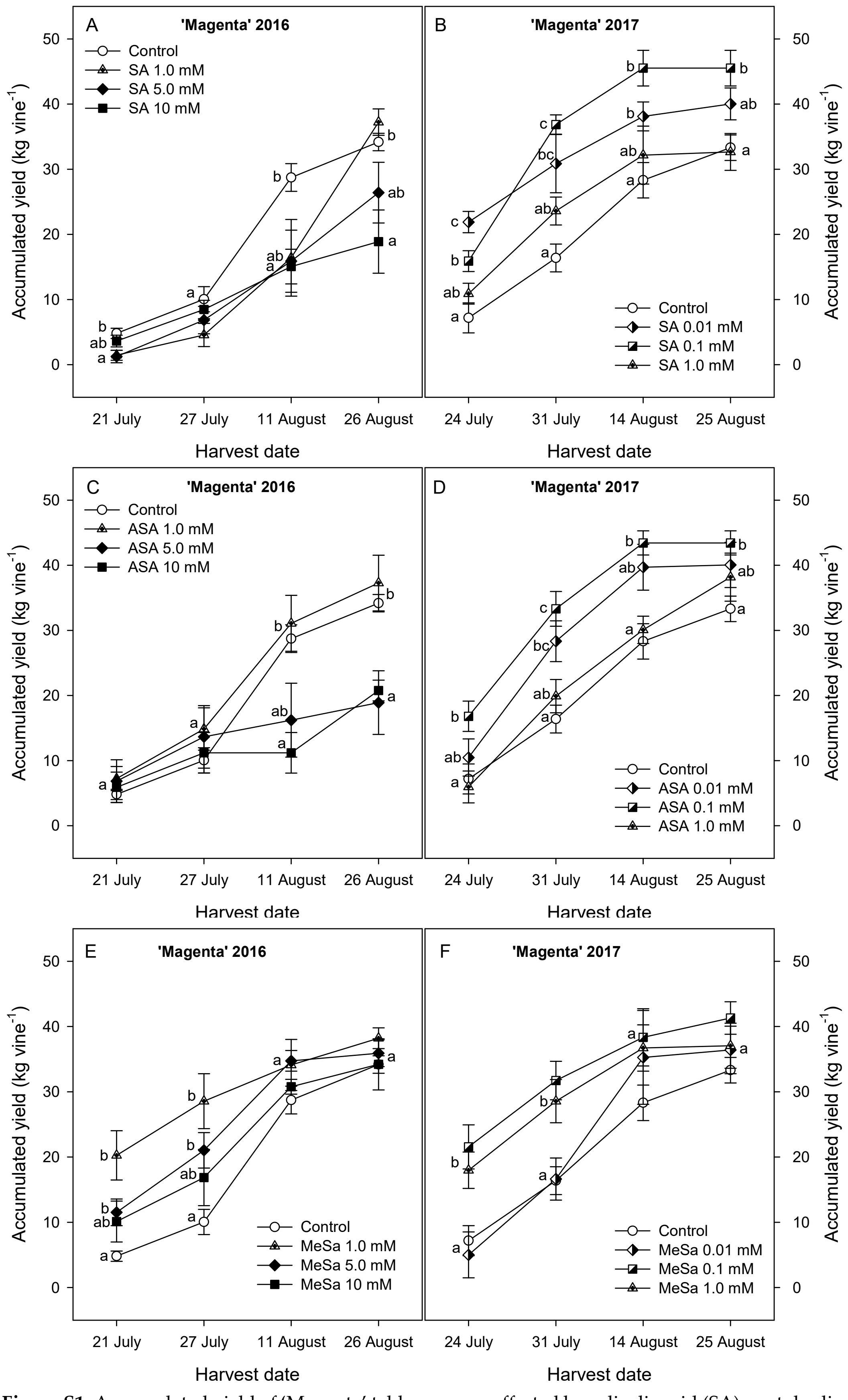

Figure S1: Accumulated yield of 'Magenta' table grape as affected by salicylic acid (SA), acetyl salicylic acid (SA) and methyl salicylate (MeSa) treatments in 2016 (A, C, E, respectively) and 2017 (B, D, F, respectively) experiments. Data are the mean ± SE of three replicates of three vines. Different letters show significant differences (*P*<0.05) among treatments for each harvest date.

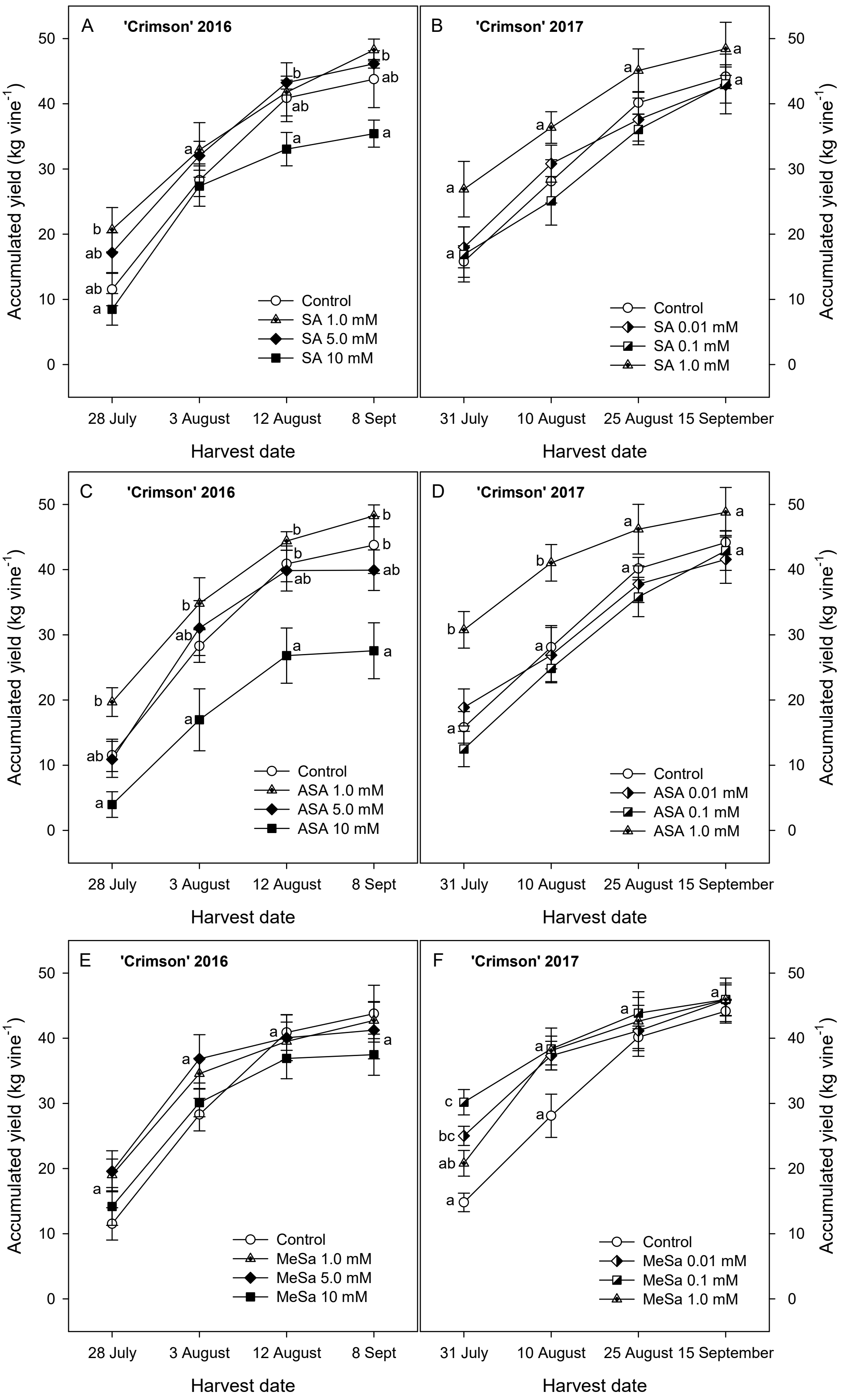

Figure S2: Accumulated yield of 'Crimson' table grape as affected by salicylic acid (SA), acetyl salicylic acid (SA) and methyl salicylate (MeSa) treatments in 2016 (A, C, E, respectively) and 2017 (B, D, F, respectively) experiments. Data are the mean ± SE of three replicates of three vines. Different letters show significant differences (P<0.05) among treatments for each harvest date.

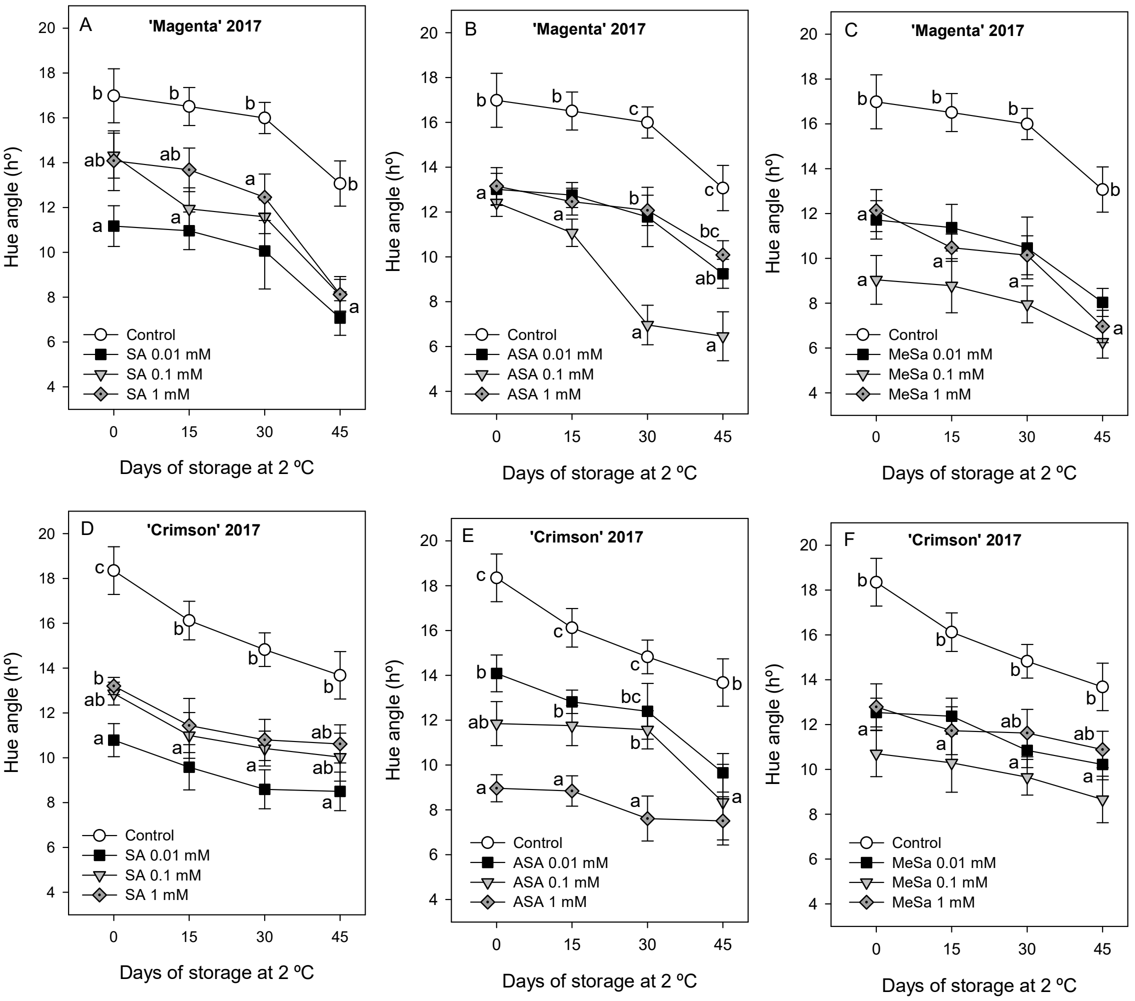

Figure S3: Effects of preharvest salicylic acid (SA), acetyl salicylic acid (SA) and methyl salicylate (MeSa) treatments on Hue angle colour evolution during storage at 2 ºC of 'Magenta' (A, B and C) and 'Crimson' (D, E and F) table grapes in 2017 experiment. Data are the mean ± SE of measures made in three replicates of 30 berries. Different letters show significant differences (*P*<0.05) among treatments for each sampling date.

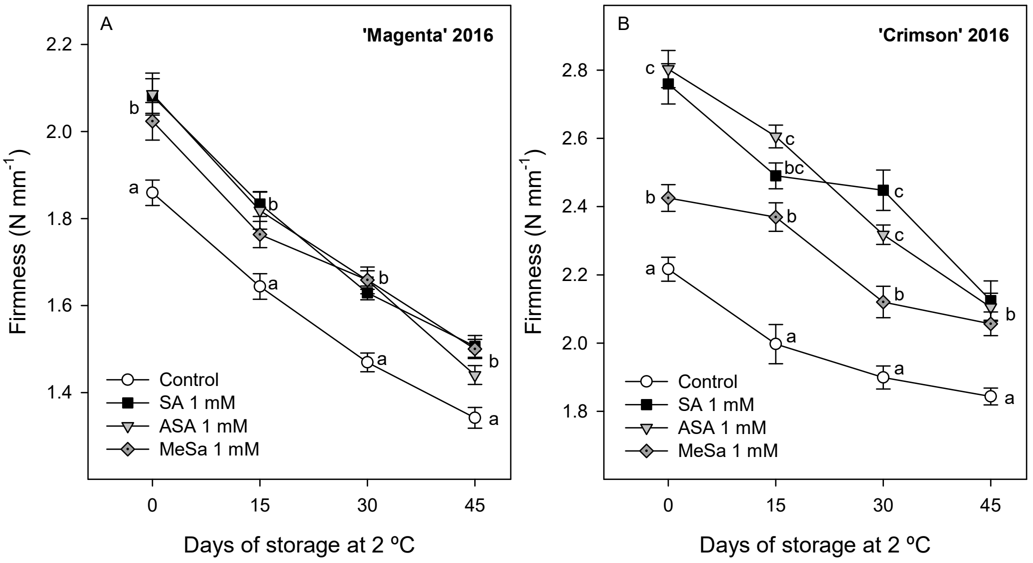

Figure S4: Effects of preharvest salicylic acid (SA), acetyl salicylic acid (SA) and methyl salicylate (MeSa) treatments on fruit firmness evolution during storage at 2 ºC of 'Magenta' (A) and 'Crimson' (B) table grapes in 2016 experiment. Data are the mean ± SE of measures made in three replicates of 30 berries. Different letters show significant differences (*P*<0.05) among treatments for each sampling date.

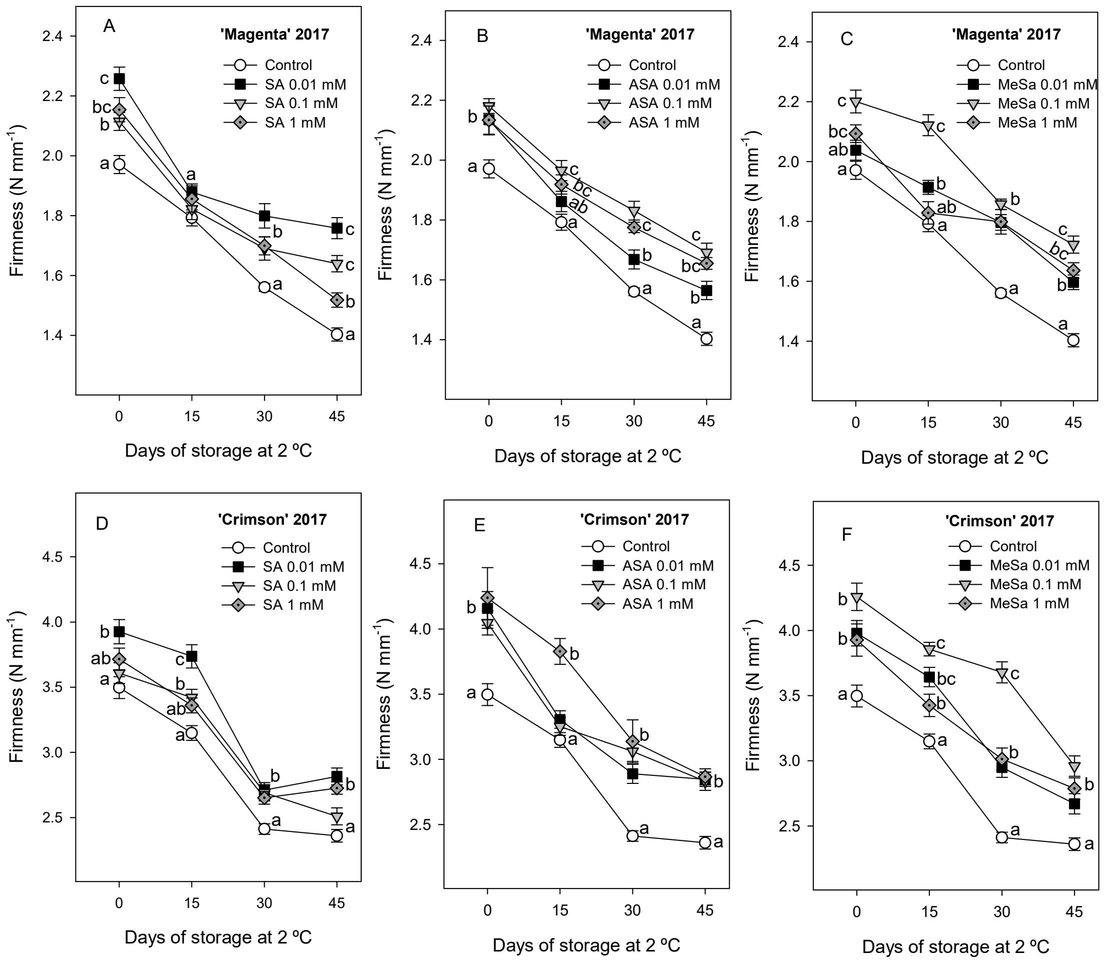

Figure 5S: Effects of preharvest salicylic acid (SA), acetyl salicylic acid (SA) and methyl salicylate (MeSa) treatments on fruit firmness evolution during storage at 2 ºC of 'Magenta' (A, B and C) and 'Crimson' (D, E and F) table grapes in 2017 experiment. Data are the mean ± SE of measures made in three replicates of 30 berries. Different letters show significant differences (*P*<0.05) among treatments for each sampling date.

Table S1: Weight loss (%) during storage in cluster of `Crimson` and `Magenta table grapes from control and salicylic acid (SA), acetyl salicylic acid (ASA) and methyl salicylate (MeSa) treated vines in 2016 and 2017 experiments.

	Days of storage at 2 °C		
	15	30	45
2016 Experiment		'Crimson'	
Control	2.74 ± 0.29abA	4.47 ± 0.42aB	7.13 ± 0.24bC
SA 1 mM	2.66 ± 0.04aA	4.33 ± 0.17aB	5.85 ± 0.16aC
ASA 1 mM	2.88 ± 0.07bA	4.59 ± 0.32aB	6.04 ± 0.39abC
MeSa 1 mM	2.74 ± 0.11abA	4.14 ± 0.19aB	5.48 ± 0.25aC
2016 Experiment		`Magenta'	
Control	3.74 ± 0.35aA	6.69 ± 0.41cB	9.09 ± 0.36bC
SA 1 mM	3.55 ± 0.21aA	6.09 ± 0.48bcB	7.42 ± 0.48aB
ASA 1 mM	3.32 ± 0.33aA	5.38 ± 0.21bB	7.51 ± 0.42aC
MeSa 1 mM	2.81 ± 0.21aA	4.51 ± 0.24aB	6.76 ± 0.38aC
2017 Experiment		'Crimson'	
Control	3.61 ± 0.09bA	5.68 ± 0.11bB	8.26 ± 0.46cC
SA 0.01 mM	2.59 ± 0.10aA	5.56 ± 0.16bB	6.33 ± 0.24aC
SA 0.1 mM	2.40 ± 0.07aA	5.75 ± 0.17bB	6.98 ± 0.04bC
SA 1 mM	2.75 ± 0.26aA	4.77 ± 0.44abB	7.07 ± 0.33abcC
ASA 0.01 mM	2.66 ± 0.30aA	4.38 ± 0.23aB	7.32 ± 0.20bcC
ASA 0.1 mM	2.50 ± 0.15aA	4.85 ± 0.39abB	6.93 ± 0.25abcC
ASA 1 mM	2.59 ± 0.23aA	4.00 ± 0.22aB	6.63 ± 0.09aC
MeSa 0.01 mM	2.91 ± 0.33abA	4.56 ± 0.52abB	7.01 ± 0.30abcC
MeSa 0.1 mM	2.07 ± 0.42aA	4.31 ± 0.81abB	6.14 ± 0.26aB
MeSa 1 mM	2.09 ± 0.39aA	4.38 ± 0.51abB	6.87 ± 0.25abC
2017 Experiment		`Magenta'	
Control	2.92 ± 0.11bA	6.73 ± 0.54cB	8.75 ± 0.28dC
SA 0.01 mM	2.29 ± 0.26abA	5.36 ± 0.62abcB	6.32 ± 0.42abcB
SA 0.1 mM	3.02 ± 0.16bA	5.37 ± 0.30abcB	7.12 ± 0.12bcC
SA 1 mM	2.86 ± 0.04bA	5.82 ± 0.41bcB	6.76 ± 0.29abcB
ASA 0.01 mM	2.50 ± 0.12aA	5.06 ± 0.32abB	7.41 ± 0.20bcC
ASA 0.1 mM	2.41 ± 0.06aA	4.87 ± 0.36abB	6.82 ± 0.12bC
ASA 1 mM	2.44 ± 0.12aA	5.54 ± 0.25bcB	7.50 ± 0.21cC
MeSa 0.01 mM	2.78 ± 0.28abA	5.24 ± 0.06bB	7.18 ± 0.26bcC
MeSa 0.1 mM	2.19 ± 0.35abA	4.60 ± 0.17aB	6.36 ± 0.07aC

MeSa 1 mM	2.76 ± 0.28abA	4.68 ± 0.14aB	6.96 ± 0.30abcC

Data are the mean ± SE of three replicates. Different capital letters show significant differences for each treatment during storage and different lowercase letters show significant differences among treatments for each sampling date at *P*< 0.05.

Table S2. Data of media maximum, minimum and medium temperatures (°C) during the growth cycle in the experimental field for 2016 and 2017 experiments. Data were recorded by an automatic weather station located close to the experimental field. *

	May		June		July		August		September	
Temperature	2016	2017	2016	2017	2016	2017	2016	2017	2016	2017
Maximum	22.12	22.37	29.24	28.44	30.63	29.61	28.76	30.52	25.80	25.56
Minimum	14.62	17.13	21.25	21.06	23.23	20.9	22.96	20.47	19.02	17.71
Medium	18.51	20.01	23.91	25.13	26.41	26.89	25.82	26.34	23.28	22.84

* Sistema de Información Agrario de Murcia. Instituto Murciano de Investigación y Desarrollo Agrario de la Región de Murcia. http://siam.imida.es/

Table S3. Comparative values of total anthocyanin concentration and total phenolic concentration on 'Crimson' and 'Magenta' table grapes from control and salicylic acid (SA), acetyl salicylic acid (ASA) and methyl salicylate (MeSa) treated vines on 2016 and 2017 experiments.

		'Crimson'		'Magenta'	
		2016	2017	2016	2017
Total anthocyanins ($g\ kg^{-1}$)	Control	0.049±0.003a	0.067±0.002b	0.035±0.003a	0.053±0.006b
	SA 1 mM	0.085±0.006a	0.097±0.007a	0.066±0.003a	0.076±0.005a
	ASA 1 mM	0.073±0.003a	0.124±0.002b	0.063±0.004a	0.084±0.006b
	MeSa 1 mM	0.096±0.007a	0.109±0.003a	0.078±0.004a	0.106±0.002b
Total phenolics ($g\ kg^{-1}$)	Control	0.777±0.016b	0.510±0.006a	0.247±0.013a	0.491±0.012b
	SA 1 mM	0.891±0.036b	0.655±0.023a	0.306±0.017a	0.577±0.023b
	ASA 1 mM	0.938±0.031b	0.643±0.023a	0.395±0.008a	0.526±0.012b
	MeSa 1 mM	0.968±0.032b	0.606±0.020a	0.475±0.022a	0.563±0.006b

* Different letters show significant differences according to Student t' test between the two growing seasons for each parameter at $P < 0.05$.
